# A Parsimonious Taxonomy of The Newly Retired: Spousal and Disability Combinations Shape Part or Complete Retirement

**DOI:** 10.3390/ijerph192013537

**Published:** 2022-10-19

**Authors:** John Rodwell, Thomas Hendry, Dianne Johnson

**Affiliations:** 1 Department of Management & Marketing, Swinburne University of Technology, Hawthorn, VIC 3122, Australia; 2 Griffith Business School, Griffith University, Brisbane, QLD 4111, Australia

**Keywords:** retirement, disability, segments, spouse retirement, health promotion, aging

## Abstract

The inadequate classification of retiree sub-groups ultimately results in misaligned policy. To generate sets of sub-groups that may be appropriately targeted for policy and interventions, variables are used that reflect the social structure of retirees, particularly the options of partial and complete retirement, marital status, gender, as well as the retirement status of the spouse, where relevant, and disability. Three sets of longitudinal Australian data were combined, each reflecting a four-year period (2003–2007, 2007–2011, 2011–2015) during which the individuals aged 45 to 69 retired (*n* = 1179). A multiway frequency analysis was performed to develop an inductive, combinatorial model of retirement from work. The resulting parsimonious taxonomy of sub-groups of the newly retired reflected main effects and interactions of key social-structural variables. Notably, a key driver of the pattern of results was that couples tend to coordinate their retirement behavior in both the decision to retire and form of retirement. Non-partnered retirees were more likely to be women. Disability was also a driver of retirement for non-partnered retirees, regardless of gender. Identifying sub-groups based on combinations of retiree characteristics can better inform policy design, appropriate health promotion interventions and potential specific triggers for enacting those policies. Overall, marital status, spousal retirement behavior and disability may each present a more useful basis for a taxonomy of retirement than more individually oriented age- and wealth-based systems.

## 1. Introduction

Effective policy is appropriately targeted when it avoids ambiguity and affects sub-groups most in need of the intervention [1]. Characteristics of older age-groups are distinct from other age cohorts. Most notably, older age groups experience a higher incidence of poor health from age-related illnesses, while often reducing their workforce participation through partial or full retirement. For older workers that remain in the workforce, health promotion programs aim to keep them happy, healthy and productive [2]. These sub-groups can be identified by distinct combinations of characteristics that occur in older age-groups. Correctly identifying these sub-groups informs health promotion programs in targeting those with known shared characteristics. For example, certain lifestyle characteristics have been associated with higher degrees of COVID-19 vaccine hesitancy, suggesting that health promotion policies could be better targeted to those households [3]. Despite the importance of targeted public health programs, studies identifying sub-groups have largely been secondary to studies that adopt a more “predictive” approach between individual factors and behavior around retirement. Some exceptions are recent studies that consider the heterogeneity of retiree characteristics as pivotal to their research [4,5,6]. These studies consider multidisciplinary factors that influence behavior and highlight the importance of understanding these dimensions collectively. Our study adopts an approach to identifying sub-groups among new retirees in Australia, which in turn can help inform health promotion policy for areas such as long-term care [6], managing complex multimorbidity [5] and eradicating senior poverty [4].

Australia is a particularly unique setting to consider retiree characteristics. First, the retirement income system in Australia is mature compared to other developed countries. Retirement savings in Australia are among the highest in the world, representing around 148% of national GDP [7], although the public means-tested Age Pension exists for retirees with lower wealth or inadequate incomes. Therefore, the decision to work during retirement is complicated by a unique set of incentives and disincentives [8]. Second, women’s workforce participation is comparatively high compared to other countries, including in older age cohorts [9,10]. However, despite increased participation in the workforce, women typically experience worse financial outcomes in retirement which affect their decisions to remain, or re-enter the workforce [11,12]. Third, the Australian healthcare system is primarily funded publicly and accessible to all retirees, regardless of income or wealth. Therefore, health concerns in retirement are partly addressed by this public “safety-net”, which is increasingly relied on by an aging population [13]. The recent introduction of the National Disability Insurance Scheme (NDIS) in Australia is particularly aimed at improving the quality of life for those with a disability [14,15]. Together, these factors suggest that characteristics of Australian retirees may differ from those in other developed countries, making the identification of the key, structural sub-groups of older citizens such as the newly retired particularly important, especially in designing appropriate health promotion interventions that could be enacted with specific triggers.

Failing to classify sub-groups appropriately also carries the risk that older age-groups will instead be grouped by broad, convenient measures, such as age and whether completely retired. A useful framework to understand the shortcomings of ignoring the heterogeneity in retirees is Elder’s well-known five life course principles. The principles of (1) development, (2) historical time and place, (3) timing, (4) linked lives and (5) agency provide insight into the complex interplay of forces that affect individual behavior over the life-cycle [16]. Clearly, when considering retirement, it is difficult to construct a single homogeneous group that addresses each principle sufficiently. For example, addressing the principle of linked lives in existing research and policy design is commonly captured in a binary single/married variable. However, as explored below, marital characteristics can be difficult to disentangle from other factors linking lives in retirement, like health and workforce participation [17,18]. Similar problems arise when considering the principle of historical time and place, with earlier research indicating that retiree characteristics differ to previous cohorts [13], and across countries [19,20]. Thus, assuming retirees are a homogeneous group with shared characteristics does not address the diversity of retirement forms.

The inadequate classification of retiree sub-groups ultimately results in misaligned policy, which may instead reflect popular age stereotypes [21,22]. If important retiree characteristics are ignored, policy may hinder rather than help the people it affects. A misalignment between structural policy and social change over time is considered structural lag [23] and while salient changes to retiree characteristics are addressed by policy changes over time, such as increased life expectancy or career longevity [24,25], other changes such as evolving spousal caregiver duties are not [26]. Identifying combinations of retiree characteristics can better inform policy design.

### 1.1. Key Structural Characteristics

To generate a useful and useable grouping of the newly retired, for whom much policy is intended, the variables used for defining the groups need to better reflect the basic social structure of retirees and of their forms of retirement. This section focuses on the variables and their combinations that have been most consistently found to shape social structure for retirees, where those variables are relatively observable and/or likely to be in government systems. Building policy on characteristics such as attitudes that are invisible or unobservable—such as satisfaction—would represent an extra degree of difficulty in forming relevant policy, when there are sufficient difficulties as it is. Consequently, this section will briefly overview retirement, particularly the options of partial and complete retirement, marital status, gender, as well as the retirement status of the spouse, where relevant, and disability or health impairment.

A particular focus of earlier research has been on the evolving patterns of employment in older age-groups. The general aging of the workforce is observable across most developed countries [13], a phenomenon partly driven by policy agendas across several western countries in the 1990’s to address a perceived labor shortage caused by older workers retiring early [27,28,29]. As such, more workers are choosing to partially retire rather than exit the workforce, abandoning or delaying the concept of full retirement [30,31]. With more older workers partially retiring, a growing body of research has focused on better understanding how these workers can remain healthy and productive [2,32], a goal that may be facilitated by better targeting of health promotion programs. 

Marital characteristics are also important with marital status associated with retirement timing [33]. In particular, many couples tend to coordinate their retirement [34,35,36], although there may be some differences by gender, with some evidence indicating that women born later are less likely to coordinate their retirement with their spouse [37]. Marital characteristics are not just relevant to retirement coordination, but also influence the form of retirement. Some examples of spousal and spouse-by-gender drivers of the structure of retirement from the literature include spouse allowances affecting workforce participation [38], spouse income affecting retirement behavior [39], husbands exhibiting more linear retirement patterns than wives [40], husbands changing retirement plans because of the financial implications of the Global Financial Crisis [41], and spouses influencing early retirement [42]. These studies indicate that marital characteristics and gender are associated with both the decision to retire, and the form of retirement adopted. The interdependence between marital characteristics and the varying forms of retirement makes retirement more of a “family affair” than an individual event [42,43].

Further, the prevalence of several disabilities increases with age [44]. For example, the prevalence of disabilities in older people across Europe and the United States has increased over time [45,46], a pattern also found in Australia where one in two people over 65 have a disability [47]. Increasingly, mental health disorders make up a larger proportion of disabilities, a phenomenon that also exists in retiree cohorts, most notably early retirees [48], although the overall prevalence of mental health disorders in retirees remains lower than the general population [49,50]. Physical disabilities are, perhaps unsurprisingly, more common in retirees. Freedman et al. [51] reports that limitations in activities of daily living affect around 12 percent of people aged over 65. 

In light of the comparatively poorer health of older age-groups, an interdisciplinary approach to retirement-focused research, for example in the fields of sociology and economics, and health-focused research has been adopted. The link between retirement and health, the so-called retirement-health nexus, has been explored in a growing body of research [52,53,54] primarily concerned with the effect of health on the timing and forms of retirement [53,55,56]. Longitudinal survey data, such as collected in the Health and Retirement Study is used in several of these studies [57,58,59]. This interdisciplinary approach is especially important given that those with disabilities are often pushed into retirement earlier than the general population [60] where they are faced with distinct challenges compared to healthy retirees, such as additional caregiving support or increased risks of abuse [61], along with increased financial stress [62]. Health in retirement is also co-dependent on other factors. For example, engaging in bridge employment has been associated with better mental health [63] while certain marital characteristics in old age have been associated with self-rated health [64]. 

Overall, the real-life representation of retiring can be summarized by inductively generating groups of retirees in terms of structural drivers of their retirement. These drivers can reflect combinations of retirement, whether partial or complete, marital status and spouse’s retirement status, which may vary by gender, as well as by disability, which in turn could be associated with the form of retirement. The inclusion of an emphasis on the consideration of couples and their potential coordination of retirement activities, strongly reflects the social structure of our lives.

### 1.2. Parsimonious Groupings to Reflect Key Structural Combinations

These characteristics—workforce participation, marital status and disability/health impairment—can be analyzed to construct a system of groupings within the older population. The context in which typologies are formed will depend on the research perspective of the given study [65]. A common approach is to posit typologies based on workforce participation derived from employment status. An early example is Gustman and Steinmeier [66] who used four types; completely retired, partially retired in the main job, partially retired outside the main job, or not retired. Constructing a taxonomy, where the data inductively elucidates the structure of sub-groups, using factors across several domains, rather than a single domain such as employment status, presents challenges. However, methods that group categorical variables, for example log linear methods, are well-suited for understanding these complex combinatorial relationships [67,68]. Since the publication of analysis of correlation tables as early as 1903 [69], the extension to comparative analysis [70] and contingency tables has become widespread over time [71], with thousands of applications noted in Beh and Lombardo’s 2014 text exploring the versatility of two-way contingency table analysis [72]. Multiway Frequency Analysis (MFA) is a particularly suitable non-parametric procedure for discrete variables, and identifies the most parsimonious group of variables to form a set of sub-groups [73,74]. Consequently, MFA will be used to generate the groupings of retirees using a parsimonious number and combination of the variables above that represent the social structure of these retirees. 

## 2. Materials and Methods

### 2.1. Sample

The data for this analysis come from the Household, Income and Labour Dynamics in Australia (HILDA) Survey, which is a large-scale, nationally representative household panel survey in Australia. Starting from 2001, HILDA annually collects information on people’s demographics, education, labor market dynamics and health status [75]. The retirement module is included in the survey every fourth year starting in wave 3 (2003). Consequently, this paper focuses on the data from waves 3, 7, 11 and 15. The data for this paper is extracted from wave 3 to wave 15 (2003 to 2015) of the first 17 waves of the survey. The University of Melbourne Ethics approval number for the HILDA survey project is 1647030.

The sample used in the analysis was composed of data matched over time in wave-pairs. Respondents indicating that they were working at time 1, who were either completely or partly retired four years later at time 2, were extracted. Top-up samples had been added to HILDA over time, especially at wave 11. Consequently, a check filter was applied whereby those respondents indicating that they completely retired at the later wave in a wave pair were limited to those who had been completely retired for less than four years. This process was applied for the three wave-pair combinations (workers at wave 3 who retired by wave 7, *n* = 368; workers in wave 7 retired by wave 11, *n* = 450; workers in wave 11, retired by wave 15, *n* = 497), for a pooled dataset of 1315 retirees who retired during each of the four-year windows. Note that only respondents 45 years of age and older were asked the key retirement questions. Further checks then led to the exclusion of one respondent who was in a nursing home, and to exclude those whose next step was often to a nursing home, which reflects a set of issues outside the scope of this paper, leaving a sample trimmed such that all were under the age of 70 at time 2, leaving 1179 respondents. Note that the analyses were repeated with an age limit of 75 and the same variables are significant, but with a higher proportion of the 70–75 year-olds moving to nursing homes, the results below focus on those less than 70 years of age so as to better inform policies associated with retirees, before some potentially move on to nursing homes.

### 2.2. Measures

The variables used to conduct the multiway frequency analysis were the socio-structural variables comprising the patterns of retirement at the time of retirement or shortly thereafter.

Gender of respondent: The variable representing the gender of the respondent was generated by matching the person number and the gender of the equivalent respondent number at time 2, the end wave of each wave pair. The responses were coded 1 male, 2 female.

Completely or Partly Retired: Respondents were asked: The next set of questions are about retirement from paid employment and your plans for retirement from paid employment. We may have already asked you about whether you are retired or not, but can I just check again: Do you consider yourself to be completely retired from the paid workforce, partly retired or not retired at all? Where the answers were: Completely retired, Partly retired, Not retired at all and Not relevant–have never been in paid work. Due to the sample selection process above, the respondents were coded as to whether they were Completely retired (1), or Partly retired (2) as at time 2, in each wave pair.

The spouse’s retired status was a variable that was coded at time 2, the end wave of each wave pair, in two stages. The respondents were initially asked: [A]t the time you [partly] retired, were you married or living in a long-term relationship with a partner? Those respondents answering No were coded as Not Married [4]. Those respondents answering Yes were asked: [W]hen you [partly] retired, was your (spouse/partner) already retired or not in the paid workforce? [1], working part-time? [2] or working full-time? [3]. The resulting variable represents the retirement status of the respondent’s spouse, if the respondent is married or in a long-term relationship, or that the respondent is not married.

The Disability variable coded whether anyone in the household had a long-term health condition, disability or impairment. Looking at [the list below], does anyone here have any long-term health condition, disability or impairment such as these? The list shown was disabilities/ health conditions which: Have lasted, or are likely to last, 6 months or more; [R]estrict everyday activity; and [C]an not be corrected by medication or medical aids. Sight problems not corrected by glasses or contact lenses, Hearing problems, [S]peech problems, [B]lackouts, fits or loss of consciousness, Difficulty learning or understanding things, [L]imited use of arms or fingers, [D]ifficulty gripping things, [L]imited use of feet or legs, [A]nervous or emotional condition which requires treatment, [A]ny condition that restricts physical activity or physical work (e.g., back problems, migraines), [A]ny disfigurement or deformity, [A]ny mental illness which requires help or supervision, [S]hortness of breath or difficulty breathing, [C]hronic or recurring pain, [L]ong-term effects as a result of a head injury, stroke or other brain damage, [A] long-term condition or ailment which is still restrictive even though it is being treated or medication is being taken for it, [A]ny other long-term condition such as arthritis, asthma, heart disease, [A]lzheimer’s disease, dementia, etc. Respondents were asked to please answer Yes (1) or No (2), which was the basis of the variable Disability. 

Years (Completely Retired): At time 2, the end wave of each wave pair, for those respondents who were completely retired, the number of years the specific respondent had been completely retired was calculated as their age at time 2 minus their age when they completely retired, in order to check that any possible duplicate cases were excluded, with some respondents part-retiring on their way to completely retiring. Respondents who part-retired and then completely retired fairly quickly within the time window were counted per their status at time 2, as being completely retired.

## 3. Results

A four-way frequency analysis was performed to develop a hierarchical log-linear model of socio-structural shapers of retirement from work. The variables analyzed were (i) whether the retiree’s spouse was working or not, or whether the retiree was single, (ii) the gender of the retiree, (iii) whether anyone in the household had a long-term health condition, disability or impairment, and (iv) whether the retiree retired completely or partly. The breakdowns of these key variables are summarized in Table 1.

The aim of the models generated via the MFA process is to find the model with the smallest number of effects that provides a fit between expected and observed frequencies, thereby parsimoniously recreating and representing the frequencies in the data [74]. The analyses were conducted on 1179 retirees and followed the process for multiway frequency analysis detailed in Tabachnick and Fidell [74]. All two-way contingency tables had expected frequencies greater than five. After the model had been finalized only one of the 32 cells was an outlier. The outlier cell contained males, with a disability in the household, whose spouse was already retired or not in the paid workforce at the time that the respondent male partly retired. The outlier cell had 27 observations, which was higher than the 17.514 cases expected from the model (standardized residual = 2.267). The model accurately represented all of the other cells.

Stepwise selection by simple deletion of effects using SPSS (version 25; Chicago, IL, USA) HILOGLINEAR produced a model that included all of the first-order effects and four of the possible six two-way associations and none of the higher-order interactions. The model had a likelihood ratio of χ^2^ (15) = 14.939, *p* = 0.456 with 95% confidence limits of 0 to 16.41, indicating a good fit between the observed frequencies in the data and the expected frequencies generated from the model. A summary of the model with results of tests of significance (partial likelihood ratio χ^2^) and their 95% confidence limits are in Table 2.

A summary of log-linear parameter estimates in raw and standardized form appears in Table 3. Disability was the strongest one-way effect, followed by spouse retired status, then retirement type, whereas the one-way effect of gender was not significant. The significant two-way effects with the strongest parameters were spouse status by gender, spouse status by retirement type, disability by retirement type, and somewhat later spouse status by disability. 

A summary of the two-way contingency tables of gender, type of retirement and disability by spouse status is shown in Table 4. The clearest distinction within the spouse status by gender two-way effect, is that there were more men who at their time of retirement had a spouse working part-time, in contrast to the two cells where there were more women who, at the time they retired, had a spouse either working full-time or they were not married. The clearest distinction within the spouse status by retirement type two-way effect is that those whose spouse was already retired were more likely to completely retire, in contrast to those retirees whose spouse was working part-time, where the retiree was more likely to partly retire.

The spouse status by disability two-way effect shown in Table 4 is the weakest of the significant two-way effects and is particularly characterized by the not married retirees being more likely to have a disability. That cell was distinct from those retirees whose spouse was working full-time having a lower rate of disability in the household and all of the other married categories having lower rates of disability.

A summary of the two-way contingency table of disability by type of retirement is shown in Table 5. The clearest distinction for the disability by retirement type two-way effect is that those with a disability in the household were more likely to then completely retire, whereas those without a disability in the household were more likely to be partly retired. There were no statistically significant two-way associations found between gender by disability, nor for gender by retirement type.

## 4. Discussion

This study constructed a parsimonious taxonomy of the newly retired using combinations of key social-structural variables that could inform public health promotion policies and activities. Perhaps the key finding emerging from the pattern of results is that couples tend to coordinate their retirement (following [34,36]), where marital characteristics and gender are associated with both the decision to retire, and the form of retirement adopted. The importance of the marital status and spousal retirement behavior presents a different basis for a taxonomy of retirement than more individually oriented systems such as Gustman and Steinmeier [66]. More generally, the strength of the effects associated with marital status may also reflect the psychosocial resources of the couple, although that more detailed emphasis could be a focus for future research.

The interdependence of the effects associated with marital status and the behavior of the spouse highlights the importance of social structures in the retirement decision and demonstrate that retirement is not only an individual-dependent event (supporting [42,43]). Marital status and spouse behavior are not just relevant to retirement coordination, but also influence the form of retirement. External forces that may be driving the strength of these effects include how spouse income affects retirement behavior [39], husbands, in the main, exhibiting more linear retirement patterns than wives [40] in terms of following social norms.

Some of the key messages emerging from the results suggest segments that may change depending on the focus of any given health promotion campaign or other public policy. For example, from a deficit lens, with more of an orientation toward illness, those older citizens going through the retirement process who are not married and have a disability represent a particular cause for concern. The complexity and real-world nature of the taxonomy is highlighted by the other effects and segments that are potentially related, but may only be partially aligned. For health support policies the not married having higher rates of disability present a certain range of issues and options, whereas from a financial policy point of view, those who are not married and female could particularly be a concern where, in Australia at least, women tend to have lower levels of wealth and independent income to retire on, which may represent a high need sub-group for programs aimed at eradicating senior poverty (per [4]). However, note that the not married and female combination is not the same as the previous (not married having higher rates of disability) combination. That is, the higher rates of disability for the not married apply irrespective of gender. Thus, the different combinations could be targeted by different policies depending on the policy purpose and issues being considered. 

Similarly, another key combination is the segment where one spouse is retired, associated with the other individual often completely retiring, particularly relative to the segment where one spouse is working part-time, and the other individual in that instance being more likely to partly retire. In those two segments, the individual appears to be following the spouse (where each member of the couple may completely retire, or where each member of the couple may partly retire). It is possible that these segments reflect recent trends in some countries, such as a couple both completely retiring for a leisure motive, giving rise to the “grey nomad” phenomenon, where retirees embark on travel activities.

This analysis highlights the need for more granularity in the segmentation of retiree pathways showing that reforms such as widespread lifting of the pension eligibility age has likely led to transfer of older worker income needs to forms of disability income support in lieu of retirement income support, rather than the assumption that workforce participation will be extended. Later retirement for people in physically intensive work, or in poorer health earlier in the life course could lead to increased financial and health detriment for those with less choice in the form of work they are able to undertake [76].

In terms of the basic effects, it is notable that the presence of disability is associated with the individual being more likely to completely retire. Although this segment may be a key driver of the effect known in the literature where those with disabilities are often pushed into retirement (per [60]), and in turn may suffer increased financial stress dependent on access to income support [62], the Australian healthcare system is relatively universal and accessible to all retirees, regardless of income or wealth. Therefore, the recently introduced National Disability Insurance Scheme (NDIS) in Australia, which is aimed at improving the quality of life for those with a disability may have a large challenge in front of them specific to some segments of retirees. Further, segments delineated by disability may present differing communication options for health promotion campaigns engaging issues such as multimorbidity [5].

The segments can also be informative from a prevention lens. For example, in seeking to retain workers in the workforce, the segments where the couple are married and both working, or married and one working, with the possible exclusion of those with a disability (if programs such as the NDIS support the disabled), campaigns could introduce interventions aimed at keeping these workers healthy and productive (applying [2,32]). In turn, such programs could impact financial issues such as pension policies and impact later health issues (and policies) due to the association between bridging employment and better health, whether mental [63] or general [64].

A more lateral segment arising from the analyses was the “outlier” cell of males with a disability in the household, whose spouse was already retired or not in the paid workforce at the time that the respondent male partly retired. The size of this cell and its basis on a combination of issues that do not fit into the general framework suggests the presence of a new social norm where males continue working part-time in retirement to support a disability among the couple. Future research could explore whether this is a new norm and/or the issues associated with this new segment.

From a more statistical perspective, the strength and presence of the combinatorial effects in the above results highlights how more “predictive”, regression-based studies may need to consider more interactions in their analyses, at least interactions that represent the social structure of retirees. A key example of the impact of the interactions are those associated with gender. The main effect of gender was not significant in the above analyses. However, gender is part of a strong two-way effect (by spouse’s retirement status). It is possible that the inconsistent findings regarding gender across studies of retirement may be due to the extent to which the two-way effect is accounted for in other variables present in those analyses. At a minimum, if the two-way effect with gender had not been included (as it often would not in many regression analyses), the one-way gender effect may have been significant. Further, that situation would be masking a potentially important segment—women who are not married—who may need to be the focus of key policy activities in future (e.g., regarding poverty in retirement). That segment would also be masked by the arguably dominant social norm of married women being more likely to be retired or working part-time, while their male spouse continues to work full-time.

The results of this study may be limited in their generalizability due to their basis in Australia. For example, Australia has relatively universal health care, which may have substantially influenced the forms of retirement found above. Similar approaches to above could be applied in other countries to develop taxonomies of retirees for their respective national context. The above results may also have been influenced by our focus on a longitudinal four-year window approach to tracking actual retirement behavior. Studies with a focus on retirement intentions could end up with different emphases, as could studies that focus on complete retirement behavior. This study focused on actual retirement behavior, whether complete retirement or part retirement so as to more comprehensively represent the behaviors of the retirees and to allow more fine-grained targeting of policy, whether health policy or financial such as pension reforms. 

## 5. Conclusions

Newly retired citizens are not one homogenous group. Effective policy can be appropriately targeted by focusing on the sub-groups most in need of the intervention [1], whether for health policy in areas such as long-term care [6], managing complex multimorbidity [5] or eradicating senior poverty [4]. The strength of the segments above reflects the importance of social structures such as the behavior of one’s spouse in a couple. The effect of the spouse was important at the one-way level and as a part of most of the two-way effects. That is, couples largely coordinate their work and retirement behavior. 

Employing a classification that reflects the observed behavior of a target group, such as retirees, can inform the alignment of policy to retiree sub-groups [21,22], thereby engaging contemporary social structures and avoiding structural lags [23]. Identifying combinations of retiree characteristics can better inform policy design. Key structural sub-groups of older citizens such as those identified above among the newly retired are particularly important in designing appropriate health promotion interventions and informing potential specific triggers for enacting those policies.

## Figures and Tables

**Table 1:** Frequencies and percentages per variable (*n* = 1179).

Effect		Count (Percentage)
Spouse Status	-Spouse Already Retired	347 (29.4%)
	-Spouse Working Part-time	216 (18.3%)
	-Spouse Working Full-time	352 (29.9%)
	-Not Married	264 (22.4%)
Disability	-Yes	442 (37.5%)
	-No	737 (62.5%)
Retirement Type	-Completely Retired	660 (56.0%)
	-Partly Retired	519 (44.0%)
Gender	-Male	578 (49.0%)
	-Female	601 (51.0%)

**Table 2:** Significance tests for hierarchical model of socio-structural drivers of retirement, *n* = 1179 over a four-year period.

Effect	df	Partial Association Chi-Square	95% CI	
			Lower	Upper
First-order effects:				
Spouse Status	3	45.76 ***	21.41	73.85
Disability	1	74.60 ***	44.59	112.31
Retirement Type	1	16.90 ***	4.63	36.86
Gender	1	0.45	0	6.87
Second-order effects:				
Spouse Status × Gender	3	85.36 ***	51.20	123.22
Spouse Status × Disability	3	24.12 ***	7.16	44.84
Spouse Status × Retirement Type	3	44.23 ***	20.32	71.86
Disability × Retirement Type	1	22.19 ***	7.56	44.49

*** *p* < 0.001, CI = Confidence Interval.

**Table 3:** Parameter estimates for hierarchical model of socio-structural drivers of retirement.

Effect	Log-Linear Parameter Estimate (Lambda)		Lambda/SE	
First-order effects:	Yes	No	Yes	No
Spouse Status [Ref. = Not Married]				
-Spouse Already Retired	0.153	−0.153	2.803	−2.803
-Spouse Working Part-time	−0.304	0.304	−4.798	4.798
-Spouse Working Full-time	0.192	−0.192	3.584	−3.584
Disability [Ref. = No disability]				
-Yes, disability	−0.267	0.267	−8.418	8.418
Retirement Type [Ref. = Partly retired]				
-Completely Retired	0.139	−0.139	4.362	−4.362
Gender [Ref. = Female]				
-Male	0.004	−0.004	0.141	−0.141
Second-order effects:	Yes	Else	Yes	Else
Spouse Status × Gender [Ref. = Not married & Male]				
-Spouse Retired & Male	0.161	−0.161	3.260	−3.260
-Spouse Working Part-time & Male	0.395	−0.395	6.519	−6.519
-Spouse Working Full-time & Male	−0.248	0.248	−4.977	4.977
Spouse Status × Disability [Ref. = Not married & Disability]				
-Spouse Retired & Disability	−0.068	0.068	−1.334	1.334
-Spouse Working Part-time & Disability	−0.001	0.001	−0.021	0.021
-Spouse Working Full-time & Disability	−0.163	0.163	−3.142	3.142
Spouse Status × Retirement Type [Ref. = Not married & Completely retired]				
-Spouse Retired & Completely Retired	0.298	−0.298	5.790	−5.790
-Spouse Working Part-time & Completely Retired	−0.260	0.260	−4.478	4.478
-Spouse Working Full-time & Completely Retired	−0.080	0.080	−1.631	1.631
Disability × Retirement Type [Ref. = No disability & Completely Retired]				
-Disability & Completely Retired	0.147	−0.147	4.616	−4.616

Ref. = referent category.

**Table 4:** Two-way contingency tables of gender, type of retirement and disability by spouse status.

Spouse Status		Male	Female	Completely Retired	Partly Retired	Disability	No Disability	Total
Spouse already retired	Count	202	145	238	109	127	220	347
	%	58.2%	41.8%	68.6%	31.4%	36.6%	63.4%	100%
Spouse working part-time	Count	149	67	91	125	78	138	216
	%	69.0%	31.0%	42.1%	57.9%	36.1%	63.9%	100%
Spouse working full-time	Count	134	218	176	176	106	246	352
	%	38.1%	61.9%	50.0%	50.0%	30.1%	69.9%	100%
Not married	Count	93	171	155	109	131	133	264
	%	35.2%	64.8%	58.7%	41.3%	49.6%	50.4%	100%
Total	Count	578	601	660	519	442	737	1179
	%	49.0%	51.0%	56.0%	44.0%	37.5%	62.5%	100%

**Table 5:** Two-way contingency table of disability by type of retirement.

		Completely Retired	Partly Retired	Total
Disability	Count	287	155	442
	% column	64.9%	35.1%	100%
No Disability	Count	373	364	737
	% column	50.6%	49.4%	100%
Total	Count	660	519	1179
	% column	56.0%	44.0%	100%

## Data Availability

The HILDA data is available and managed by the Melbourne Institute of Applied Economic and Social Research at The University of Melbourne (https://melbourneinstitute.unimelb.edu.au/hilda). The release 19 data were used to conduct the analyses initially. The statistics were checked using the wave 20 release of the relevant data.
